# Warming Accelerates the Onset of the Molecular Stress Response and Increases Mortality of Larval Atlantic Cod

**DOI:** 10.1093/icb/icac145

**Published:** 2022-09-21

**Authors:** Rebekah A Oomen, Halvor Knutsen, Esben M Olsen, Sissel Jentoft, Nils Chr Stenseth, Jeffrey A Hutchings

**Affiliations:** Centre for Ecological and Evolutionary Synthesis (CEES), Department of Biosciences, University of Oslo, 0371 Oslo, Norway; Center for Coastal Research (CCR), Department of Natural Sciences, University of Agder, 4604 Kristiansand, Norway; Institute of Marine Research, Nye Flødevigveien 20, 4817 His, Norway; Department of Biology, Dalhousie University, Halifax, NS B3H 4J1, Canada; Center for Coastal Research (CCR), Department of Natural Sciences, University of Agder, 4604 Kristiansand, Norway; Institute of Marine Research, Nye Flødevigveien 20, 4817 His, Norway; Center for Coastal Research (CCR), Department of Natural Sciences, University of Agder, 4604 Kristiansand, Norway; Institute of Marine Research, Nye Flødevigveien 20, 4817 His, Norway; Centre for Ecological and Evolutionary Synthesis (CEES), Department of Biosciences, University of Oslo, 0371 Oslo, Norway; Centre for Ecological and Evolutionary Synthesis (CEES), Department of Biosciences, University of Oslo, 0371 Oslo, Norway; Center for Coastal Research (CCR), Department of Natural Sciences, University of Agder, 4604 Kristiansand, Norway; Center for Coastal Research (CCR), Department of Natural Sciences, University of Agder, 4604 Kristiansand, Norway; Institute of Marine Research, Nye Flødevigveien 20, 4817 His, Norway; Department of Biology, Dalhousie University, Halifax, NS B3H 4J1, Canada

## Abstract

Temperature profoundly affects ectotherm physiology. Although differential thermal responses influence fitness, thus driving population dynamics and species distributions, our understanding of the molecular architecture underlying these responses is limited, especially during the critical larval stage. Here, using RNA-sequencing of laboratory-reared Atlantic cod (*Gadus morhua*) larvae of wild origin, we find changes in gene expression in thousands of transcripts consistent with a severe cellular stress response at both ambient and projected (+2°C and +4°C) temperatures. In addition, specific responses to stress, heat, and hypoxia were commonly identified in gene ontology enrichment analyses and 33 of the 44 genes comprising the minimum stress proteome of all organisms were upregulated. Earlier onset of the stress response was evident at higher temperatures; concomitant increased growth and mortality suggests a reduction in fitness. Temporal differences in gene expression levels do not correspond to differences in growing degree days, suggesting negative physiological consequences of warming beyond accelerated development. Because gene expression is costly, we infer that the upregulation of thousands of transcripts in response to warming in larval cod might act as an energetic drain. We hypothesize that the energetically costly stress response, coupled with increased growth rate at warmer temperatures, leads to faster depletion of energy reserves and increased risk of mortality in larval cod. As sea surface temperatures continue to rise over the next century, reduced fitness of Atlantic cod larvae might lead to population declines in this ecologically and socioeconomically important species. Further, our findings expand our understanding of transcriptomic responses to temperature by ectothermic vertebrate larvae beyond the critical first-feeding stage, a time when organisms begin balancing the energetic demands of growth, foraging, development, and maintenance. Linking the molecular basis of a thermal response to key fitness-related traits is fundamentally important to predicting how global warming will affect ectotherms.

## Introduction

Temperature has profound effects on ectotherm physiology, impacting metabolic and developmental rates (Clarke and Fraser 2004), aerobic scope (Clark et al. 2013), and inducing cellular stress responses (Schulte 2014). The resulting phenotypic and fitness consequences ultimately drive population dynamics and species distributions (Pörtner 2002; Schulte et al. 2011). Despite clear links to individual fitness and population viability, the molecular mechanisms underlying thermal plasticity are poorly understood (Logan and Buckley 2015; Oomen and Hutchings 2017). This is especially true of early life stages, which encompass critical periods for survival and development and are particularly sensitive to temperature (Pörtner and Farrell 2008). Ectothermic larvae typically tolerate a narrower range of temperatures than later life stages and these tolerances often more accurately reflect the climatic boundaries within which species successfully colonize (Abele 2012). Further, the influence of temperature during development has consequences for thermal tolerances and acclimation capacities later in life (Scott and Johnston 2012). Thus, understanding the effects of temperature during early development at the molecular level is critical for determining the physiological limitations of ectotherms and predicting their individual and population responses to global climate change.

Most investigations of responses to thermal extremes at the molecular level have narrowly focused on heat shock proteins (Chen et al. 2018). Advances in transcriptomics of non-model species (i.e., those which lack genomic tools and resources) have broadened the interpretation of thermal responses beyond the heat shock response to the highly complex genome-wide suite of processes that affect an organism's performance near the edges of their thermal range (Logan and Buckley 2015; Kingsolver and Woods 2016). While energetically costly heat shock proteins have been shown to improve short-term survival, sustained overexpression can have trade-offs, reducing the energy available for growth, development, and reproduction (Tomanek 2010). Therefore, the extent and duration of gene and protein expression in response to sub-lethal thermal stress, and associated fitness consequences, can have profound impacts on ectotherm populations experiencing climate warming (Kingsolver and Woods 2016).

Knowledge of how the transcriptomic thermal response impacts and interacts with growth and development of ectotherms in early life is limited; most studies have been undertaken on juveniles and adults (Moyano et al. 2017). Among transcriptomic studies of early life-stage thermal plasticity in ectothermic vertebrates, few have extended beyond the stage of first feeding, the critical point at which organisms must begin to balance the energetic demands of foraging, growth, development, and maintenance. Microarray studies investigating responses to warming in (pre-feeding) fish larvae have had limited time points (*n* = 2) and do not link variation in gene expression to survival (Long et al. 2012; Meier et al. 2014), despite fitness measures being critical to the interpretation of how thermal responses affect individuals and populations.

Nonetheless, these studies have revealed dysregulation of thousands of genes in response to temperature increases of 3**°**C (Meier et al. 2014) and 6**°**C (Long et al. 2012). For example, in a study of larval zebrafish (*Danio rerio*) 96 h post-fertilization, differential expression of 2860 temperature-responsive genes was coupled with inhibitory effects of thermal extremes on larval development (Long et al. 2012). Seemingly contrary evidence from the invertebrate realm suggests that 2**°**C of warming accelerates normal developmental transcription patterns in larvae of the coral *Acropora palmata*, although the fitness implications are unclear (Polato et al. 2013).

We hypothesize that small temperature increases concordant with those predicted to occur over the next century (IPCC 2013) are likely to cause vast shifts in gene expression, associated fitness and, consequently, population viability. Here, we use RNA-sequencing to investigate the effects of ambient and projected global shifts in temperature (+2**°**C and + 4**°**C) on genome-wide expression patterns during early development of Atlantic cod (*Gadus morhua*), a widely distributed marine eurytherm with a thermally and developmentally sensitive pelagic larval phase. A comprehensive experimental design, comprising several temperatures and time points (beyond the critical stage of first feeding), allows us to characterize the transcriptomic response to warming in larval fish and evaluate the relationship between the thermal response and developmental time. Further, we quantify growth and survival reaction norms to understand the fitness implications of the transcriptomic response. In doing so, the present study contributes to our understanding of the physiological limitations of ectotherms during early life and provides a resource for functional studies of temperature-responsive genes during early development.

## Materials and methods

### Experimental design

**Fig. 1:**
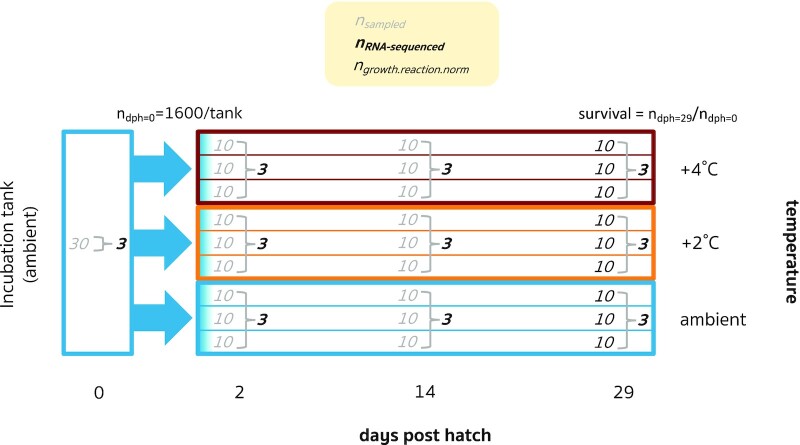
Experimental design. Newly hatched larvae were transferred to independent experimental tanks assigned to three temperatures with three replicate tanks per temperature and reared for 29 days post hatch. The ambient temperature was 9°C. The number of larvae sampled from each tank and, of these, RNA-sequenced and used for the growth reaction norm analysis is shown.

We conducted RNA sequencing of larval Atlantic cod that were reared at three temperatures in the laboratory to assess variation in gene expression, growth, and survival with temperature over time (Fig. 1). Sixty-six adult Atlantic cod were collected near the *Institute of Marine Research, Flødevigen* on the Norwegian Skagerrak coast (58.39603N, 8.73322E) in December 2012. All cod were allowed to spawn undisturbed in a 45 m^3^ spawning basin at the *Institute of Marine Research, Flødevigen*, from February to May 2013. Cod were held at ambient temperature and photoperiod and fed shrimp daily until the end of the spawning period, when they were sacrificed by a blow to the head prior to dissection for sex identification and fin tissue collection.

Midway through the spawning season, fertilized eggs were collected and incubated at 9°C in a 900 L flow-through seawater tank until hatch, when they were randomly distributed among 40 L rearing tanks with flow-rates of 0.35 L/min (i.e., ∼2 h turnover rate). Larvae were reared at three temperatures (9°C, 11°C, and 13°C) with three replicate tanks per temperature initially containing 1600 larvae each (Fig. 1). These temperatures represent the ambient seawater temperature outside the Flødevigen facility, in the vicinity where the adult cod were collected, and a 2°C and 4°C increase representing projected climate scenarios by the year 2100 (IPCC 2013). The temperatures in the rearing tanks were 9°C upon transfer from the incubation tank and then gradually changed to the experimental temperatures over the course of 24 h. Larvae were reared under a constant light intensity of 2000 lux and fed *Brachionus plicatilis* rotifers enriched with RotiGrow *Plus*™ (Reed Mariculture, USA) in excess (4500 prey/L three times daily, at approximately 10:00 am, 2:00 pm, and 6:00 pm). Water temperatures were recorded daily and water quality parameters (oxygen, pH, and ammonia concentration) were monitored with no notable deviations.

Ten larvae from each of the nine tanks were randomly sampled at 2, 14, and 29 dph, and an additional 30 larvae were sampled from the hatching tank at 0 dph (Fig. 1). Larvae were individually placed in RNAlater™ (an ammonium sulphate solution) on a glass slide and immediately photographed, using a stereoscope with a Leica DFC 425 C Camera. Samples were preserved in RNAlater™ at -20°C prior to DNA and RNA extraction.

Standard length at 29 dph was measured from the photographs, using ImageJ (Abràmoff et al. 2004), and considered a proxy for growth rate, following Hutchings et al. (2007). Survival was quantified as the number of larvae alive in each tank at 29 dph relative to the number initially in each tank. This count was obtained prior to that day's sampling, but was not otherwise adjusted for sampling mortality.

After 29 dph, the larvae continued to be reared with a change in feed: a 1:1 mixture of rotifers and *Artemia* from 32–39 dph and *Artemia* only from 40–43 dph. Survival was measured again at 43 dph to assess the consistency of temperature effects over time, beyond the end of the larval stage. Gene expression was not measured beyond 29 dph due to the confounding effect of different feed types with size and temperature; therefore, we refer to day 29 as the end of the experiment.

### DNA/RNA isolation

Of the sampled larvae, 30 were selected for RNA-seq: three from each of the three temperature treatments and three time points, plus three samples from the hatching tank (0 dph) to serve as a baseline (Fig. 1; see Supplementary Table S3 for details). Samples were selected from two of the tank replicates, which were considered to be biological replicates given the lack of phenotypic variation among tanks (random effect *σ*^2^ = 0 in the growth model). Larvae were dissected into proximal (including the head, organs, and part of the tail) and distal (the remainder of the tail) sections, which were used for RNA and DNA isolations, respectively. DNA was extracted from adult and larval tissue, using the E-Z 96 Tissue DNA Kit (Omega Bio-tek) in accordance with the manufacturer's instructions. Parentage analysis using methods described by Roney et al. (2018) identified three full-sib/two half-sib families derived from two fathers and three mothers, with 90% of offspring coming from the same parent pair (Table S3).

### RNA isolation, sequencing, and assembly

For each larva, total RNA was isolated using the RNeasy Mini Kit (Qiagen) according to the manufacturer's instructions with the following specifications. Tissue homogenization was carried out using a Fastprep-24 Instrument (MP Biomedicals) for 30 s at 4.0 M/S in 1.5 mL tubes containing ceramic beads and 250 μL 1x lysis buffer. RNA was eluted in two steps using 25 μL of RNase-free water each. Quality of RNA isolates was evaluated using an Agilent 2100 Bioanalyzer (BioRad) before and after library preparation with the TruSeq^TM^ RNA low-throughput protocol (Illumina) and a fragmentation time of 4 min.

Libraries were sequenced with a 100 bp paired-end (PE) protocol on the Illumina HiSeq 2500 platform at the Norwegian Sequencing Centre (www.sequencing.uio.no), producing a total of 798 million read pairs. Trimming and adapter removal were performed on all sequences (Oomen et al. 2022), reducing the dataset to 740 million read pairs. Transcriptome assembly was performed *de novo* using the Trinity software suite v.2014–07–17 (http://trinityrnaseq.github.io) and annotated using Trinotate v.2.0.1 (https://trinotate.github.io). See Oomen et al. (2022) for assembly details and comparisons of the *de novo* assembly and reference genome-based assemblies.

### Differential expression statistical analysis

Differential expression analysis was carried out using edgeR v.3.16.5 (Robinson et al. 2010) in R v.3.3.2 (R Core Team 2014). We filtered transcripts that did not have a count per million (CPM) >1 in at least three samples. Pairwise comparisons were then carried out between all samples compared to the baseline sample, as well as across time within temperatures, and across temperatures within times, using a false discovery rate (FDR)-corrected *P*-value of 0.05. Venn diagrams were constructed using BioVenn (Hulsen et al. 2008) and Venny (http://bioinfogp.cnb.csic.es/tools/venny/) to compare differentially expressed gene lists among pairwise comparisons.

### Gene ontology enrichment analysis

GO enrichment analysis and network construction were performed using ClueGO v.2.3.2 (Bindea et al. 2009) and BiNGO v.3.0.3 (Maere et al. 2005) in Cytoscape v.3.2.1 (Shannon et al. 2003) to identify significantly enriched biological processes involved in the thermal response. Annotations were compared to a human (in ClueGO) or custom (i.e., constructed from the Blastx hits from Trinotate; in BiNGO) gene ontology database. Up- and down-regulated genes were analyzed separately for both analyses and only the GO category “Biological Processes” was analyzed as we considered it to be the most phenotypically informative. The Gene Fusion option was used in ClueGO to enhance clustering. GO terms identified by ClueGO were clustered into medium-specificity upper-level GO categories using a kappa-scoring algorithm based on gene membership similarity. Upper-level clustering in ClueGO facilitates interpretations of large numbers of enriched GO terms for those genes with annotations (in the form of gene names) that match the human database, while the custom GO database implemented in BiNGO identifies enriched processes using all available GO annotations (in the form of a numerical GO ID). An FDR-corrected *P*-value of 0.05 and otherwise default parameters were used for both analyses.

### Downstream analysis of *de novo*-assembled transcripts

Due to the ability to annotate novel loci and an apparently greater sensitivity for detecting likely biologically meaningful differential expression in our experiment (e.g., greater average GO enrichment relative to the numbers of differentially expressed genes), we chose a *de novo* assembly approach (Oomen et al. 2022). The numbers and log-fold changes of genes that were differentially expressed when compared to the baseline were evaluated relative to developmental time using the growing degree day metric, which is the sum of daily temperatures measured above a temperature threshold (in this case 0°C) that accounts for the relationship between temperature and rates of enzymatic reactions (Neuheimer and Taggart 2007). We performed a post-hoc evaluation for the presence of the minimum stress proteome (a set of 44 universally conserved stress proteins; Kültz 2005) in our experiment by manually searching the transcriptome annotations for gene descriptions corresponding to those contained in Kültz (2005) Table 1 and observing whether these transcripts were differentially expressed relative to the baseline.

**Table 1:** Transcripts annotated to genes that comprise part of the minimum stress proteome that were differentially expressed (FDR >0.05) relative to the baseline in larval Atlantic cod reared at three temperatures.

Transcript ID	Gene name	Description	Min. E value	D2-13°C	D14-9°C	D14-11°C	D14-13°C	D29-9°C	D29-11°C	D29-13°C
DNA damage sensing/repair										
c79008_g1	MLH1	DNA mismatch repair protein MLH1	1.00E−126			6.62		5.07		
c139074_g1	MSH6	DNA mismatch repair protein Msh6	0	5.96		8.08		6.47		
c143511_g1	MSH2	DNA mismatch repair protein Msh2	0	6.28		8.18		7.15		
c79247_g1	RA51A	DNA repair protein RAD51 homolog A	0	5.54		8.31		6.53		
c141131_g1	TOP1	DNA topoisomerase 1	0	6.67		9.58		9.07		
c185971_g1	TOP3A	DNA topoisomerase 3-alpha	0	4.75		6.21		4.93		
Energy metabolism										
c136064_g1	AT2C1	Calcium-transporting ATPase type 2C member 1	0	5.58		6.72		5.42		
c145796_g2	AT2B1	Plasma membrane calcium-transporting ATPase 1	6.00E−42						−1.47	
c150107_g1	AT2B3	Plasma membrane calcium-transporting ATPase 3	0	6.98		8.17		7.01		
c153430_g1	AT2A1	Sarcoplasmic/endoplasmic reticulum calcium ATPase 1	2.00E−54			−1.18				
c153430_g4	ATC	Calcium-transporting ATPase sarcoplasmic/endoplasmic reticulum type	3.00E−54	6.48		6.72		5.70		
c153660_g13	AT2B3	Plasma membrane calcium-transporting ATPase 3	1.00E−147						−1.36	
c155386_g2	AT2B1	Plasma membrane calcium-transporting ATPase 1	0	7.47		8.61		7.73		
c157062_g2	AT2A1	Sarcoplasmic/endoplasmic reticulum calcium ATPase 1	2.00E−98			−0.98				
c158484_g6	AT2A1	Sarcoplasmic/endoplasmic reticulum calcium ATPase 1	4.00E−174			−1.18				
c160449_g5	AT2A1	Sarcoplasmic/endoplasmic reticulum calcium ATPase 1	6.00E−64			−1.25				
c130005_g1	CISY	Citrate synthase	0	8.57		9.89		7.85		
c149079_g1	ACLY	ATP-citrate synthase	0	7.83		9.41		6.47		
c159927_g2	ACLY	ATP-citrate synthase	0					−0.96	−1.17	
c141763_g1	ENOA	Alpha-enolase	0	9.59		10.59		9.19		
c140798_g1	PGM2	Phosphoglucomutase-2	0	5.85		7.94		6.00		
c141603_g1	PGM5	Phosphoglucomutase-like protein 5	9.00E−61					−1.64		
c263700_g1	PGM1	Phosphoglucomutase-1	0	7.64		8.28				
Fatty acid/lipid metabolism										
c133422_g2	PATL1	Protein PAT1 homolog 1	5.00E−24	4.97		7.13		5.44		
c110543_g1	ACSL4	Long-chain-fatty-acid—CoA ligase 4	4.00E−142			6.59		5.30		
c129849_g1	ACSL5	Long-chain-fatty-acid—CoA ligase 5	0	5.68		6.68				
c134376_g1	ACSL1	Long-chain-fatty-acid—CoA ligase 1	0	5.61		6.99				
c143921_g1	ACSL3	Long-chain-fatty-acid—CoA ligase 3	0	6.99		8.46		6.98		
c79025_g1	DHB4	Peroxisomal multifunctional enzyme type 2	0	6.74		8.04		6.19		
Molecular chaperones										
c101741_g1	DJC11	DnaJ homolog subfamily C member 11	4.00E−115	5.58		6.75				
c106402_g1	DNJB2	DnaJ homolog subfamily B member 2	4.00E−34	5.73		7.06		5.32		
c111354_g1	DNJC5	DnaJ homolog subfamily C member 5	1.00E−27	5.27		6.16				
c116500_g1	DNJC2	DnaJ homolog subfamily C member 2	2.00E−64	6.08		7.61		5.89		
c118111_g1	DNAJ1	DnaJ protein homolog 1	4.00E−103	6.80		8.79		6.93		
c118596_g1	DJB11	DnaJ homolog subfamily B member 11	1.00E−151	6.08		8.17		7.07		
c120618_g1	DNJC7	DnaJ homolog subfamily C member 7	6.00E−136	6.28		7.94		6.01		
c122274_g1	DNJA1	DnaJ homolog subfamily A member 1	6.00E−150	7.35		8.70		7.46		
c123966_g1	DNJC3	DnaJ homolog subfamily C member 3	2.00E−121	5.62		7.76		5.80		
c125071_g1	DNJC9	DnaJ homolog subfamily C member 9	1.00E−46	5.43		8.83		7.22		
c136250_g1	DJB12	DnaJ homolog subfamily B member 12	1.00E−72	5.68		7.07		6.02		
c137180_g1	DJC16	DnaJ homolog subfamily C member 16	1.00E−54	5.36		6.99		5.04		
c141320_g1	DJC10	DnaJ homolog subfamily C member 10	0	5.33		6.87		6.60		
c144998_g2	DJC27	DnaJ homolog subfamily C member 27	0					−0.81		
c149091_g2	DNJC4	DnaJ homolog subfamily C member 4	6.00E−53			1.08				
c149153_g5	DNJC5	DnaJ homolog subfamily C member 5	2.00E−98			−0.71				
c53913_g1	DNJA2	DnaJ homolog subfamily A member 2	5.00E−104	7.17		9.01		6.71		
c79127_g1	DNJB2	DnaJ homolog subfamily B member 2	4.00E−41	4.87		7.99		6.39		
c138398_g2	HSP70	Heat shock 70 kDa protein	6.00E−101						2.02	
c158105_g1	HSP70	Heat shock 70 kDa protein	8.00E−50			3.90	3.91	3.38		
c159822_g1	HSP70	Heat shock 70 kDa protein	3.00E−70			3.33				
c160682_g2	HSP70	Heat shock 70 kDa protein	1.00E−142			3.17				
c237034_g1	GRPE1	GrpE protein homolog 1	2.00E−44	5.87		6.79				
c144731_g1	PPWD1	Peptidylprolyl isomerase domain and WD repeat-containing protein 1	0			7.14		6.24		
Other functions										
c128565_g1	IMPA1	Inositol monophosphatase 1	1.00E−123			−2.01				−1.84
c138690_g1	IMPA3	Inositol monophosphatase 3	3.00E−47	4.94		6.94		5.96		
c143207_g1	IMPA1	Inositol monophosphatase 1	5.00E−143		2.32	2.96	2.32	2.90	3.10	2.94
c171140_g1	IMPA2	Inositol monophosphatase 2	2.00E−81	5.44		6.62				
c114902_g1	NDK7	Nucleoside diphosphate kinase 7	1.00E−110	5.49		6.92		6.21		
Protein degradation										
c131456_g1	GABT	4-aminobutyrate aminotransferase	5.00E−172	4.74		6.70		5.88		
c17068_g1	YME1	ATP-dependent zinc metalloprotease YME1 homolog	0	6.19		8.30		5.16		
c285460_g1	LONM	Lon protease homolog	0	6.29		8.21		5.73		
c143333_g1	PPCE	Prolyl endopeptidase	0	4.32		6.83		5.26		
c139221_g3	MASP1	Mannan-binding lectin serine protease 1	7.00E−131					−1.13		
c141329_g1	YM67	Putative serine protease K12H4.7	3.00E−97	6.38		7.62				
c157193_g1	MASP1	Mannan-binding lectin serine protease 1	2.00E−33						2.39	
Redox regulation										
c119300_g1	DDH1	2-hydroxyacid dehydrogenase homolog 1	3.00E−77	8.18		8.65		7.09		
c55314_g1	6PGD	6-phosphogluconate dehydrogenase, decarboxylating	0	7.49		8.50				
c131734_g1	ALDH2	Aldehyde dehydrogenase	0	8.28		8.66		6.36		
c146004_g1	AL7A1	Alpha-aminoadipic semialdehyde dehydrogenase	0	7.65		8.61				
c147930_g3	AL8A1	Aldehyde dehydrogenase family 8 member A1	8.00E−69	−1.75						-1.90
c147930_g5	AL8A1	Aldehyde dehydrogenase family 8 member A1	0	−1.88						
c160121_g1	A16A1	Aldehyde dehydrogenase family 16 member A1	5.00E−43	7.46		8.91		6.08		
c160121_g3	BETB	NAD/NADP-dependent betaine aldehyde dehydrogenase	3.00E−25	4.97		6.14				
c84031_g1	MMSA	Methylmalonate-semialdehyde dehydrogenase [acylating]	0	7.66		8.50				
c85116_g1	AL3A2	Fatty aldehyde dehydrogenase	2.00E−157	7.05		7.33				
c127863_g1	GPDA	Glycerol-3-phosphate dehydrogenase [NAD(+)]	1.00E−139	6.51		7.63				
c152592_g5	GPDA	Glycerol-3-phosphate dehydrogenase [NAD(+)]	5.00E−52			-0.99	−1.22			
c80177_g1	GPDM	Glycerol-3-phosphate dehydrogenase	0	6.22		7.43		5.87		
c142412_g1	IDH3B	Probable isocitrate dehydrogenase [NAD] subunit beta	2.00E−124	6.77		7.69		6.52		
c142481_g1	IDHP	Isocitrate dehydrogenase [NADP]	0	9.19		10.51		9.13		
c31815_g1	IDH3A	Probable isocitrate dehydrogenase [NAD] subunit alpha	0	7.10		8.40		7.00		
c41378_g1	IDH3G	Isocitrate dehydrogenase [NAD] subunit gamma	1.00E−135	7.48		9.16		7.36		
c87013_g1	IDHC	Isocitrate dehydrogenase [NADP] cytoplasmic	3.00E−74	6.94		8.41		6.34		
c87013_g2	IDHC	Isocitrate dehydrogenase [NADP] cytoplasmic	5.00E−142	8.38		9.56		7.62		
c82288_g1	MSRA	Peptide methionine sulfoxide reductase MsrA	2.00E−67	5.97		6.18				
c101226_g1	PRDX6	Peroxiredoxin-6	1.00E−98	5.61		6.72				
c129981_g1	PRDX1	Peroxiredoxin 1	3.00E−105	7.52		7.77		5.67		
c290185_g1	PRDX4	Peroxiredoxin-4	2.00E−106	5.94		6.43				
c60400_g1	PRDX5	Peroxiredoxin-5	3.00E−51	8.03		8.25		6.07		
c142815_g1	PROD	Proline dehydrogenase 1	2.00E−146	7.00		7.06				
c100326_g1	NU1M	NADH-ubiquinone oxidoreductase chain 1	5.00E−52	11.08	10.43	13.11	9.69	11.44	9.37	9.49
c104850_g1	QOR	Quinone oxidoreductase	5.00E−118	5.24		6.71				
c126329_g1	NDUS1	NADH-ubiquinone oxidoreductase 75 kDa subunit	0	7.67		8.83		7.82		
c129954_g1	NU5M	NADH-ubiquinone oxidoreductase chain 5	1.00E−56	10.32	9.45	12.17	8.57	10.42	7.88	8.23
c136868_g1	ETFD	Electron transfer flavoprotein-ubiquinone oxidoreductase	0	6.62		8.52		7.18		
c154835_g7	SQRD	Sulfide:quinone oxidoreductase	0					0.91	1.11	
c133346_g1	SODC	Superoxide dismutase [Cu-Zn]	1.00E−31	7.59		9.54				
c23313_g1	SODM	Superoxide dismutase [Mn]	1.00E−100	6.70		8.13		5.83		
c236807_g1	SODM	Superoxide dismutase [Mn]	3.00E−81	8.80		9.41				
c238857_g1	SODC	Superoxide dismutase [Cu-Zn]	4.00E−30	7.51		9.09				
c106726_g1	TRX1	Thioredoxin-1	9.00E−36	7.33		8.32		6.36		
c108197_g1	THIOM	Thioredoxin	5.00E−39	5.80		7.60		5.84		
c109589_g1	TRX1	Thioredoxin-1	3.00E−28	8.36		7.07		5.49		
c124927_g1	TMX1	Thioredoxin-related transmembrane protein 1	6.00E−37	6.66		7.74		5.66		
c125785_g1	TXND9	Thioredoxin domain-containing protein 9	1.00E−65	5.83		7.87		6.71		
c133642_g1	PLP3B	Thioredoxin domain-containing protein PLP3B	2.00E−27			6.35		5.05		
c133860_g1	TXNL1	Thioredoxin-like protein 1	5.00E−87	5.25		6.76		4.44		
c286827_g1	TXND5	Thioredoxin domain-containing protein 5	3.00E−94	4.88		6.59				
c7693_g1	TMX2B	Thioredoxin-related transmembrane protein 2-B	1.00E−57	5.04		6.77		5.33		

Only contrasts in which differential expression was detected are included.

### Growth and survival statistical analysis

The effect of temperature on growth was evaluated in R v.3.3.2 (R Core Team), using a linear mixed-effects model with temperature as a fixed effect and tank nested within temperature as a random effect. Survival was modeled in relation to temperature as a fixed effect, using a generalized linear model with a quasi-binomial distribution to account for overdispersion (i.e., dispersion parameter >1; in this case, 3.54 and 2.00 for days 29 and 43, respectively). Back-transformed model estimates were used for plotting survival reaction norms. The assumptions of normality and homogeneity of variances were not violated except for a single datum from the low temperature treatment in the survival analyses, which we chose to retain.

## Results

### Differential gene expression between each temperature at each time point relative to the baseline

**Fig. 2:**
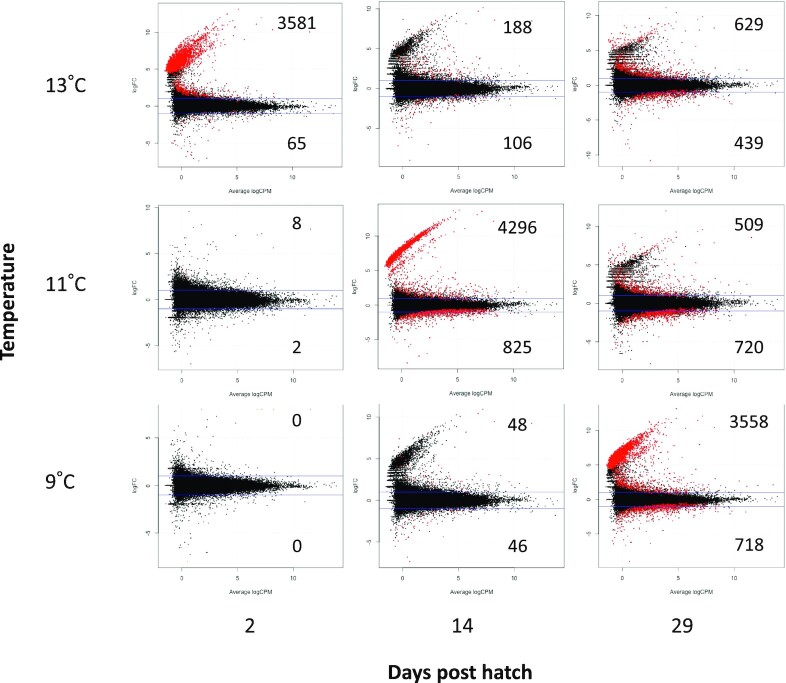
Differential gene expression of larval Atlantic cod (*G. morhua*) at ambient temperature, +2°C and +4°C. Log fold-change *vs*. average log counts-per-million of transcripts compared between each sample group (temperature × time) and the baseline sample (0 dph). The number of significantly up- and down-regulated transcripts are given in the upper and lower right-hand corners, respectively, and indicated in red based on a FDR-corrected *P*-value of 0.05. Blue lines represent positive and negative 2-fold differences in expression.

We raised laboratory-hatched cod larvae of wild origin at three temperatures (9°C, 11°C, and 13°C). We sampled a total of three larvae across two tank replicates at each temperature at 2, 14, and 29 days post hatch (dph), and an additional three larvae from the hatching tank at 0 dph (prior to transfer to temperature treatments) to serve as a baseline sample (*n* = 30). Differential expression analysis of 51,075 *de novo* assembled transcripts revealed that genomic expression varied among time points and temperatures, with a time-by-temperature interaction (Fig. 2; Supplementary Material Table S1; for assembly details, see Oomen et al. 2022). At 2 dph, no difference in expression was observed at 9°C relative to the baseline sample taken prior to transfer (0 dph at 9°C), indicating a lack of response to transfer or major change in development over this brief time period. At the same time point, 10 and 3646 differentially expressed genes were detected at 11°C and 13°C, respectively, suggesting a minimal response to a short-term 2°C increase in temperature, but a comparatively substantive response to a short-term 4°C increase. At 14 dph, the number of differentially expressed genes increased to 94 at 9°C and 5121 at 11°C, while they declined to 294 at 13°C. At 29 dph, the greatest number of differentially expressed genes was observed at 9°C (4276), although many were still detected at higher temperatures (1229 and 1068 at 11°C and 13°C, respectively).

**Fig. 3:**
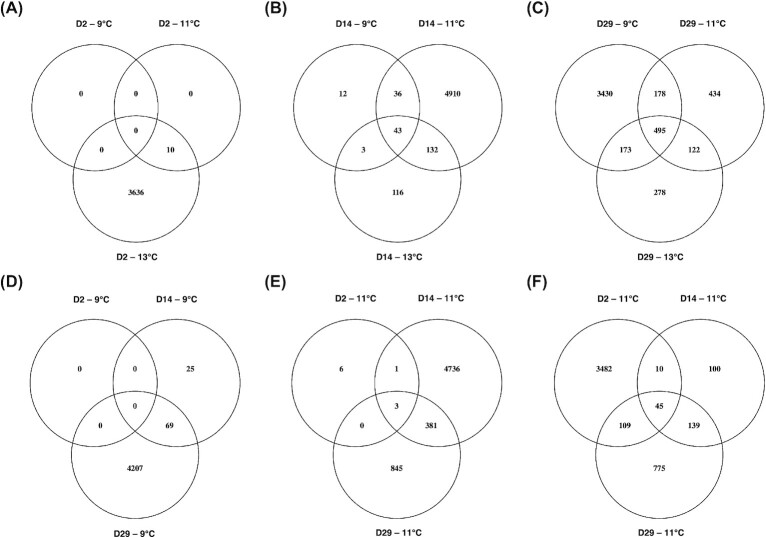
Overlap of differentially expressed genes. Venn diagrams depicting the overlap of differentially expressed (FDR <0.05) genes among temperature treatments at (A) 2 dph, (B) 14 dph, and (C) 29 dph, and among time points at (D) 9°C, (E) 11°C, and (F) 13°C.

There was substantial overlap among temperatures at which genes were differentially expressed relative to the baseline. At 2 dph, all 10 genes that were differentially expressed at 11°C were also differentially expressed at 13°C (Fig. 3A), representing a common transcriptomic response to short-term warming. At 14 dph, most of the differentially expressed genes at 9°C were also detected at 11°C, and many of these were also detected at 13°C (Fig. 3B), perhaps representing a common developmental trajectory. Substantial numbers of differentially expressed genes were common between each set of temperatures (617–673) and among all three temperatures (495) at 29 dph (Fig. 3C).

There were differences between time points in which genes were differentially expressed relative to the baseline. Relatively little overlap between 2 and 14 dph at 13°C (55/294 genes at 14 dph; Fig. 3E) suggests a major shift in transcription across this time period. Many new genes were differentially expressed at 29 dph relative to earlier time points, while some were also common to those detected at 14 dph (all temperatures) and 2 dph (13°C only) (Fig. 3D-F).

### Differential expression between temperatures

Direct tests for differential expression between temperatures within time points confirm that short-term exposure to 11°C (2°C increase) had little or no detectable impact on gene expression levels, while short-term exposure to 13°C (4°C increase) upregulated thousands of genes (Supplementary Table S1). After 2  weeks of exposure, 1507 and 2324 genes were upregulated at 11°C relative to 9°C and 13°C, respectively, while only four differentially expressed genes were detected between 9°C and 13°C. There were essentially no detectable differences in expression between temperatures after 4 weeks of exposure, suggesting that the responses to long-term exposure to different temperatures were similar.

### Differential expression over time

Direct tests for differential expression between time points within temperature treatments indicate a gradual shift in expression at 9°C, with a greater change in expression during the latter half of the experiment and the majority of differentially expressed genes detected only in pairwise tests between 0/2 and 29 dph (Table S1). The numbers of up- and downregulated genes both increased over time, but more genes were upregulated overall. A drastic shift in expression was observed between 2 and 14 dph at 11°C, with nearly twice as much upregulation as downregulation. This was followed primarily by downregulation between 14 and 29 dph, where the number of differentially expressed genes was about half as many as between the previous time points. At 13°C, expression largely decreased between 2 and 14 dph, then remained relatively constant between 14 and 29 dph.

### Temporal shift in common transcriptomic response

The differential expression analyses described herein specify a transcriptomic response characterized by vast upregulation that occurs earlier at higher temperatures, followed by downregulation of a portion of this response (except at 9°C, for which no time point subsequent to the peak of the response was available). This is exemplified by the greatest differences in gene expression relative to the baseline being observed at D2-13°C (i.e., 2 dph and 13°C), D14-11°C, and D29-9°C. The majority (2448) of these differentially expressed genes overlapped among all three responses, while 985 and 891 additionally overlapped between D29-9°C and D14-11°C, and D14-11°C and D2-13°C, respectively (Supplementary Fig. S1a). The GO terms associated with these differentially expressed genes overlapped even more, with 5902/8655 common among all three samples (Supplementary Fig. S1b). This suggests earlier onset of a common transcriptomic response as temperature increases, along with some temperature- or time-specific gene expression.

**Fig. 4:**
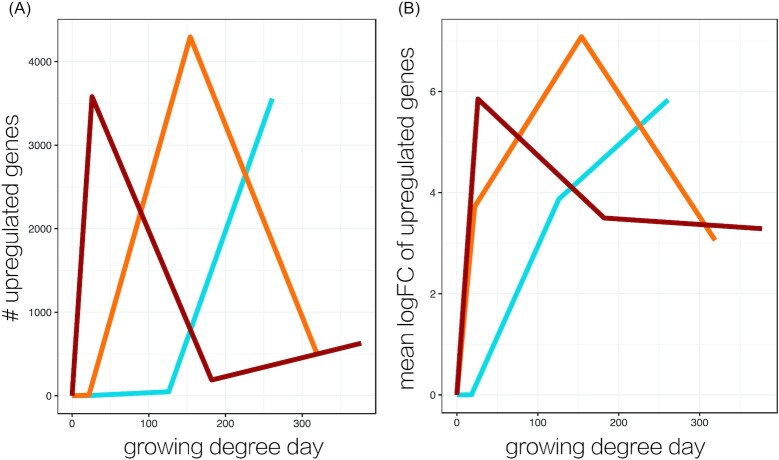
Gene upregulation by growing degree day. (A) Number of upregulated genes and (B) mean logFC of upregulated genes relative to the baseline sample (0 dph), plotted according to the growing degree day (i.e., 1 degree × 1 day) at which the sample was collected.

Importantly, these temporal differences in gene expression levels do not correspond to differences in growing degree days. The magnitude of the common transcriptomic responses, in terms of both the numbers of differentially expressed genes and their log fold-changes, peaked at different degree days (Fig. 4), in addition to different experimental time points (Fig. 2).

### GO enrichment analysis

The number of significantly enriched GO terms differed between two enrichment methods. Enrichment with BiNGO (Maere et al. 2005) tended to be more sensitive than ClueGO (Bindea et al. 2009) when enrichment was generally low (e.g., <50 BiNGO terms), but the opposite was true when enrichment was high (Supplementary Table S2). Yet, these methods showed similar temperature trends at each time point, both in terms of the numbers of enriched GO terms and their associated functions (Supplementary Table S2).

After 2 days of exposure, the few upregulated genes at 11°C were enriched for cellular respiration according to BinGO, as were the genes upregulated at 9°C after 14 days of exposure (Supplementary data 1).

**Fig. 5:**
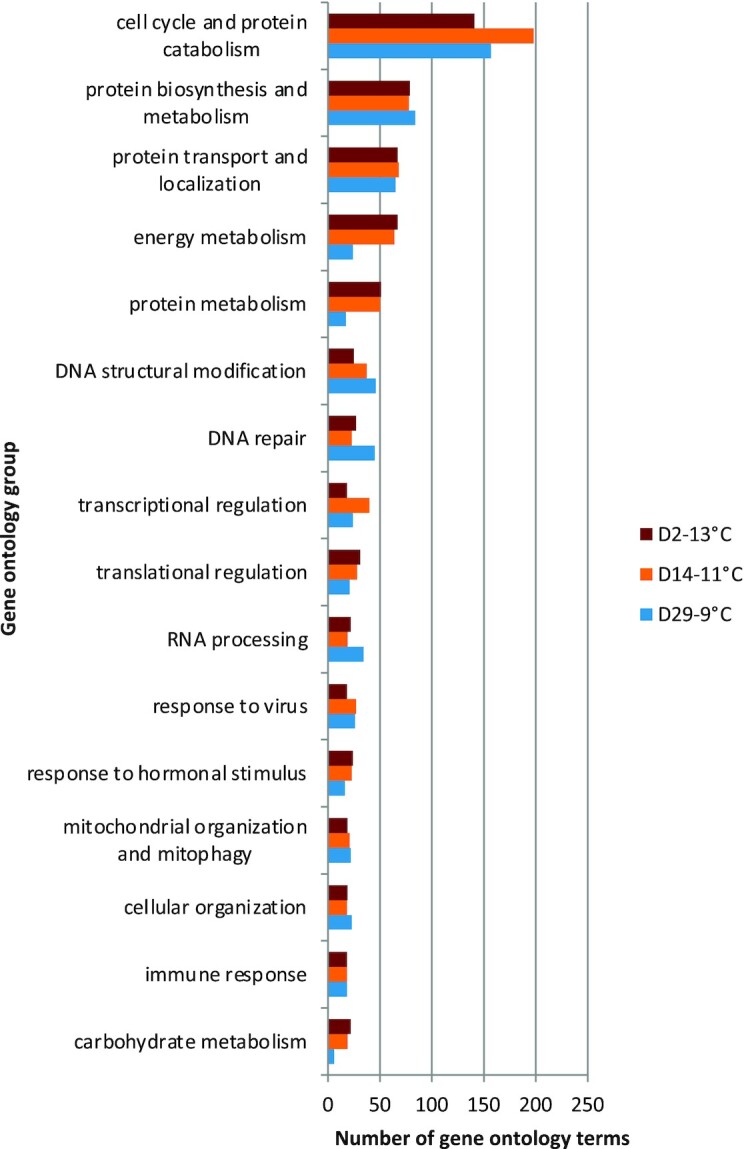
Biological processes represented by the GO groups collectively containing the greatest number of enriched (FDR <0.05) GO terms based on ClueGO analysis of the Trinity transcripts upregulated (FDR <0.05) at D2-13°C, D14-11°C, and D29-9°C. The GO groups are manually defined across separate analyses for each sample based on a high degree of overlap among terms (see Supplementary Fig. S2).

Genes that were upregulated during the peak of the common transcriptomic response (i.e., at D2-13°C, D14-11°C, and D29-9°C) were enriched for 305,334, and 307 (BiNGO) and 1545, 1756, and 1595 (ClueGO) GO terms, respectively (Supplementary Table S2). Most of the BiNGO-enriched terms were associated with development and oogenesis, cellular respiration and metabolism (particularly lipid metabolism), gene expression regulation, cell cycle activity, and protein folding, metabolism, and localization (Supplementary data 1). Within this common response, the number of terms related to the cell cycle decreased with temperature, whereas the number of terms related to energy and protein metabolism increased with temperature. The same ClueGO-enriched GO category was the largest category at D2-13°C, D14-11°C, and D29-9°C, consisting of 141–198 enriched GO terms (Fig. 5). Most of these were involved in cell cycle regulation (e.g., cell cycle checkpoint [GO: 75], regulation of cell cycle [GO: 51,726]), including cell cycle arrest (GO: 7050,71,156) and protein metabolism (e.g., protein catabolic process [GO: 30,163], proteolysis [GO: 6508]). Other GO terms within this upregulated enrichment group were associated with a response to hypoxia (GO: 36293, 36294, 61418, 70482, 71453, 71456), antigen presentation (GO: 2474, 19884, 48002), and autophagy (GO: 6914, 10506).

Among the next largest GO enrichment groups observed at the peak of the common transcriptomic response were those dominated by protein biosynthesis, metabolism, transport and localization, energy metabolism, including cellular respiration, chromatin and histone modification, DNA repair, regulation of transcription and translation, responses to hormonal stimuli, cellular/mitochondrial organization and autophagy/mitophagy, and adaptive immune and viral responses (Fig. 5). These GO groups were represented by similar numbers and types of GO terms at D2-13°C, D14-11°C, and D29-9°C, although there were fewer terms associated with metabolic processes at D29-9°C. Further, a large GO group associated with brain and nervous system development at D29-9°C (25 terms, Group 194) was absent from the warmer temperatures (although 4 [D2-13°C] and 6 [D14-11°C] of these terms were enriched in clusters related to other processes), and a group of heart developmental processes was larger at D29-9°C (20 terms) compared to D2-13°C and D14-11°C (8 terms each).

Among these commonly upregulated responses at D2-13°C, D14-11°C, and D29-9°C, those enriched GO terms unique to D2-13°C included more terms related to metabolism and energy production, whereas those unique to D29-9°C included more terms related to development, cell cycle processes, and the immune system (Supplementary data 2). Those enriched GO terms unique to D14-11°C contained intermediate amounts of metabolic and cell cycle-related terms relative to the other treatments. The numbers of enriched GO terms for upregulated genes associated with the endoplasmic-reticulum-associated protein degradation (ERAD) pathway (11, 15, and 22) and apoptotic signalling pathways (10, 16, and 22) increased with temperature for D29-9°C, D14-11°C, and D2-13°C, respectively. Enriched GO terms specifically related to oxidative stress were only present at D2-13°C (4) and D14-11°C (6). Genes upregulated at D2-13°C, D14-11°C, and D29-9°C were characterized by similar numbers of enriched GO terms related to cell cycle arrest (2–3), neuron death (3–4), cellular responses to heat (2–5), hypoxia (7–8), and stress in general (9–11). In contrast, the numbers of enriched GO terms explicitly associated with the immune system (31, 25, and 19) and the Wnt signalling pathways (14, 9, and 3) decreased with temperature at D29-9°C, D14-11°C, and D2-13°C, respectively.

Upregulated genes at D14-13°C, D29-11°C, and D29-13°C (i.e., time points following the peak of the response at each temperature) were primarily enriched for developmental and immune-related processes (Supplementary data 1 and 2). At day 29, these included BinGO-enriched terms related to heart development (e.g., GO: 55,009), the acute inflammatory response (GO: 2541), and the regulation of a suite of interleukins (GO: 32653, 32673, 32674, 32693, 32714, 32753). ClueGO-enriched terms were mainly associated with brain (GO: 21762, 30901) and heart (11°C only) development.

Downregulated genes at D2-11°C and D2-13°C were largely enriched for light perception, while at D14-9°C they were primarily enriched for cardiac development and muscle contraction (Supplementary data 3). Processes related to development and visual perception continued to be the most commonly enriched processes among downregulated genes in all treatments for the duration of the experiment (Supplementary data 3 and 4).

### Minimum stress proteome

Of the 44 genes that comprise the minimum stress proteome (Kültz 2005), 40 were detected in our *de novo* transcriptome annotation database. Thirty-three of these were differentially expressed in at least two of the three temperature treatments (at D2-13°C, D14-11°C, and/or D29-9°C), with the fewest detected at D29-9°C (Table 1). Many genes were represented by multiple transcripts and most were upregulated across treatments.

### Growth and survival reaction norms

**Fig. 6:**
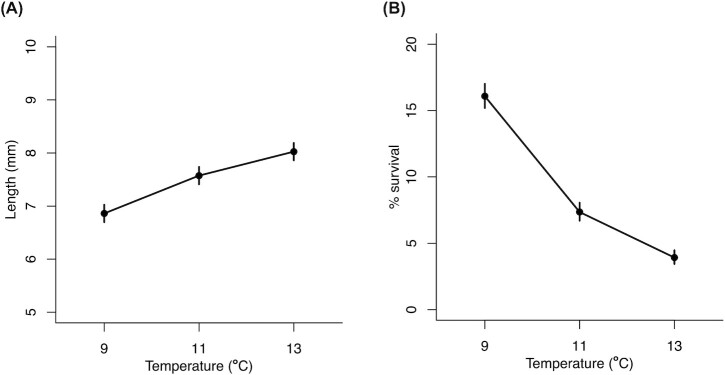
Thermal reaction norms for larval Skagerrak Atlantic cod. (A) Length and (B) survival at 29 dph.

Thermal reaction norms at 29 dph show that growth increased with temperature (*F*_2,87_ = 12.7, *P* < 0.001; Fig. 6A) while survival decreased (*P* < 0.001; Fig. 6B). The magnitudes of these effects were substantial: a 117% increase in growth and a 75% reduction in survival from 9°C to 13°C. The negative effect of increased temperature on survival was also apparent at 43 dph (79% reduction from 9°C to 13°C; *P* < 0.001; Supplementary Fig. S3).

## Discussion

Here we show that warmer temperatures accelerate the onset of a broad change in transcription in larval Atlantic cod while increasing growth and mortality. Aside from temporal differences, a similar pattern of gene transcription consistent with a severe cellular stress response was observed at all temperatures by the end of the larval stage, including the local ambient temperature (9°C) that has already risen an average of 1°C since the late 1980s (Barceló *et al*. 2016; Supplementary Fig. S4) and projected temperatures under future warming scenarios of + 2°C (11°C) and + 4°C (13°C) (IPCC 2013). Therefore, current and future sea temperatures in the Skagerrak Sea are above the threshold for thermal stress when larvae are exposed chronically in a laboratory setting. Our findings suggest that recent climate change might already be negatively impacting the productivity of the depleted Skagerrak cod populations and that future climate change is expected to exacerbate these effects in a nonlinear manner.

### Transcriptomic response to warming

Warming generally increases metabolic energy requirements and oxygen consumption (Fry and Hart 1948). Indeed, transcripts involved in cellular respiration were the earliest to be upregulated at all temperatures in our experiment, and enrichment of this process increased with temperature at the peak of the common transcriptomic response. Sufficient warming causes a build-up of oxygen metabolism byproducts, reactive oxygen species, that damages complex molecules and cellular structures (Abele and Puntarulo 2004). Damage to macromolecules activates the highly conserved cellular stress response, the core components of which include (1) the protection of macromolecules (e.g., through chaperoning of misfolded or damaged proteins and DNA repair mechanisms), (2) regulation of the cell cycle to temporarily arrest cell proliferation, (3) metabolic shifts and, if the stress is sufficiently severe, and (4) programmed death (apoptosis) and removal of terminally damaged cells (Kültz 2005). Overall, these processes dominate the common transcriptome we observed. The enrichment of GO terms specific to stress in general and the response to heat are consistent with a broad and severe thermal stress response at all temperatures in the present study. The enrichment of GO terms related to hypoxia (oxygen deficiency) is also consistent with warming-related stress, as the decreased oxygen solubility with temperature coupled with increased oxygen consumption widens the gap between supply and demand. Our inference of a stress response is further supported by the upregulation of 33 of the 44 genes comprising the minimum stress proteome of all organisms (Kültz 2005). A similar number (31 of 44) of these stress genes were upregulated in yeast exposed to diverse stressors (Gasch et al. 2000). The lack of the full set in our experiment might be attributable to incomplete assembly, constitutive expression, or regulation by processes other than transcription (e.g., post-translational modification or protein turnover).

The majority of differentially expressed transcripts in our experiment were upregulated relative to the baseline, similar to that observed in larval zebrafish following acute exposure to heat (Long et al. 2012). High enrichment for upregulation of protein biosynthesis, regulation of transcription and translation, and RNA processing likewise suggest activation of a large suite of cellular machinery that is likely costly to maintain (Wagner 2005).

In addition to earlier onset of the stress response at higher temperatures, there is some evidence that the severity of the stress response increased with temperature. Greater enrichment for upregulated genes involved in oxidative stress and protein metabolism, particularly the ERAD pathway, suggests a greater build-up of reactive oxygen species and damaged proteins at higher temperatures (Slimen et al. 2014). The associated increase in apoptotic signalling pathways (including those specifically induced by endoplasmic reticulum stress) is consistent with more prevalent terminal cell damage at higher temperatures. Similarly, genes involved in oxidative stress-induced neuron death were upregulated only at the warmer temperatures. Greater enrichment of upregulated genes related to carbohydrate metabolism and energy production at 11°C and 13°C might represent a larger metabolic shift in response to stress, a thermodynamic consequence related to the positive relationship between temperature and metabolism, or both.

Developmental transcription also differed markedly between temperatures. Many processes related to visual perception and development were commonly downregulated over time, consistent with the completion of some organ development and optical system formation. However, the lack of processes related to nervous system and heart development among genes upregulated at warmer temperatures suggests a disruption of neural and cardiac development. The reduction in enrichment of upregulated genes involved in the Wnt signalling pathways at warmer temperatures is also consistent with altered developmental progress. These highly conserved and strictly controlled pathways perform critical functions in cell proliferation and differentiation in developing tissues and the immune system, and their dysregulation is implicated in developmental abnormalities and malignancies (Staal et al. 2008). Coral (*A. palmata*; Polato *et al*. 2013) and zebrafish (Long *et al*. 2013) larvae upregulate genes involved in Wnt pathways in response to heat and cold stress, respectively, suggesting that Wnt-associated genes might be widely vulnerable to thermal disruptions.

### Earlier onset of the transcriptomic response

We found that the transcriptomic stress response occurred earlier at higher temperatures, to an even greater extent than can be accounted for by developmental time (i.e., growing degree days). The growing degree day metric is widely applied to developing ectotherms, based on the essentially linear relationship between acclimation temperature and metabolic rate across the majority of the thermal window (Neuheimer and Taggart 2007). Because the growing degree day relationship follows a normal developmental rate, temporal deviations in transcription from this trend are consistent with thermal effects on gene expression that are in addition to potential effects of temperature-mediated development. Further, the differences in growth rates among temperatures in our experiment are small compared to the differences in the timing of transcription. However, the relationships between thermal rates at different levels of biological organization are unclear and likely extremely complex, making comparisons difficult (Chaui-Berlinck et al. 2004). Nonetheless, acceleration of normal developmental transcription such as that documented in coral larvae over 72 h of exposure to just 2°C of warming (Polato et al. 2013), also has potential fitness consequences.

### A potential mechanistic link to fitness

Thermal stress depletes energy reserves such as lipid stores (Klepsatel et al. 2016), and the molecular response to thermal stress is energetically costly in particular. For example, continued overexpression of HSPs is known to negatively impact growth rate and fertility (Tomanek 2010). Such exhausting expression might help to explain why performance and its upper thermal range decrease with increasing duration of exposure to even nonlethal temperatures (Schulte et al. 2011). This hypothesis is supported by models, based on thermal performance curves for ingestion rate, that incorporate the energetic costs associated with producing heat shock proteins in Tobacco hornworm (*Manduca sexta*) larvae (Kingsolver and Woods 2016). It is reasonable to infer that the upregulation of thousands of transcripts that we observed in response to warming in larval cod might act as a similar energetic drain, as gene expression is costly (Wagner 2005).

Faster growth, as observed at higher temperatures in the present study and in marine fish larvae generally (Pepin 1991), can improve fitness by reducing the duration of the vulnerable larval stage (Anderson 1988). Yet, fast growth can be suboptimal due to conflicts in energy allocation (Billerbeck et al. 2001). If fast-growing fish are unable to sufficiently reallocate energy away from growth in response to stress, the high energetic demands of the molecular stress response might exceed energy availability. Accordingly, mortality increased with temperature in our experiment. Thus, we hypothesize that the energetically costly stress response, coupled with increased growth rate at warmer temperatures, leads to faster depletion of energy reserves and increased risk of mortality in larval cod. Such energetic limitations are likely to be exacerbated in the wild, where food is limited compared to the laboratory environment, especially at warmer temperatures (Rogers et al. 2011).

### Future research directions

The ambient temperature in our experiment is likely to be experienced during the latter, warmer part of the regional spawning period (January–May) in a relatively southern part of the distribution of this cold-water adapted species. Additional experiments are needed to characterize larval developmental expression at colder temperatures that are likely to be experienced earlier in the spawning season and farther north. Further, thermal responses can vary among populations adapted to different thermal regimes (Oomen and Hutchings 2015a). Genetic variation in thermal plasticity has been shown for growth and survival of cod larvae in the northeast Atlantic (Oomen and Hutchings 2015b, 2016). Therefore, further research is needed to determine how the transcriptomic response observed in our study might differ between cod populations. Measures of protein levels would help to further elucidate the mechanistic link between gene transcription and fitness in thermal responses, as would measures of energy usage (e.g., oxygen consumption, lipid storage levels).

### Potential implications

As sea surface temperatures continue to rise over the next century, reduced fitness of Atlantic cod larvae might lead to population declines in this ecologically and socioeconomically important species. Indeed, the physiological consequences of 2–4°C of warming observed in the present study contribute to a mechanistic understanding of the negative association between temperature and recruitment for relatively warm-water cod populations inhabiting regions such as coastal Skagerrak (Rogers and Stenseth 2017) and the North Sea (O'Brien et al. 2000). Accelerated development at warmer temperatures might lead to reduced population connectivity by shortening the duration of the pelagic larval phase, thereby limiting dispersal distance. Rapid adaptation to warmer temperatures might also occur given the apparently strong selection pressure observed in the present study, but only if heritable variation in thermal responses exists (Nussey et al. 2005).

## Supplementary Material

icac145_Supplemental_Files

## Data Availability

The genomic data underlying this article will be made available in the European Nucleotide Archive, where they will be associated with this publication. They will also be made available upon request.

## Notes

Sadly, the co-author Jeffrey A. Hutchings died prior to submission of a revised version of this manuscript. He is deeply missed.
