# Supplementary material for: Warming Accelerates the Onset of the Molecular Stress Response and Increases Mortality of Larval Atlantic Cod
Source: Integr Comp Biol. 2022 Sep 21;62(6):1784–801. doi: 10.1093/icb/icac145 (PMC9801969; doi:10.1093/icb/icac145)
Supplement: icac145_Supplemental_Files [file icac145_supplemental_files.zip › icb-2022-0143-File009.docx]

## Supplementary Materials

**Supplementary Tables**

Table S1: Numbers of transcripts differentially expressed in pairwise tests. Number of up- (+), down- (-), and dys- (+/-) regulated transcripts detected (A) at each temperature at each time point relative to the baseline sample, (B) between temperature treatments within time points, and (C) between time points within temperature treatments.

| Day post hatch | Temperature (°C) | Direction | # DE transcripts |
| --- | --- | --- | --- |
| *a) All vs. baseline (0 dph at 9°C)* | |  |  |
| 2 | 9 | + | 0 |
|  |  | - | 0 |
|  |  | +/- | 0 |
|  | 11 | + | 8 |
|  |  | - | 2 |
|  |  | +/- | 10 |
|  | 13 | + | 3581 |
|  |  | - | 65 |
|  |  | +/- | 3646 |
| 14 | 9 | + | 48 |
|  |  | - | 46 |
|  |  | +/- | 94 |
|  | 11 | + | 4296 |
|  |  | - | 825 |
|  |  | +/- | 5121 |
|  | 13 | + | 188 |
|  |  | - | 106 |
|  |  | +/- | 294 |
| 29 | 9 | + | 3558 |
|  |  | - | 718 |
|  |  | +/- | 4276 |
|  | 11 | + | 509 |
|  |  | - | 720 |
|  |  | +/- | 1229 |
|  | 13 | + | 629 |
|  |  | - | 439 |
|  |  | +/- | 1068 |
| *b) Temperature effects* | |  |  |
| 2 | 9-11 | + | 0 |
|  |  | - | 0 |
|  |  | +/- | 0 |
|  | 11-13 | + | 3291 |
|  |  | - | 64 |
|  |  | +/- | 3355 |
|  | 9-13 | + | 3556 |
|  |  | - | 49 |
|  |  | +/- | 3605 |
| 14 | 9-11 | + | 1507 |
|  |  | - | 30 |
|  |  | +/- | 1537 |
|  | 11-13 | + | 156 |
|  |  | - | 2324 |
| Day post hatch | Temperature (°C) | Direction | # DE transcripts |
|  |  | +/- | 2480 |
|  | 9-13 | + | 4 |
|  |  | - | 0 |
|  |  | +/- | 4 |
| 29 | 9-11 | + | 0 |
|  |  | - | 0 |
|  |  | +/- | 0 |
|  | 11-13 | + | 1 |
|  |  | - | 0 |
|  |  | +/- | 1 |
|  | 9-13 | + | 0 |
|  |  | - | 0 |
|  |  | +/- | 0 |
| *c) Time effects* |  |  |  |
| 0*-2 | 9 | + | 0 |
|  |  | - | 0 |
|  |  | +/- | 0 |
| 2-14 |  | + | 50 |
|  |  | - | 36 |
|  |  | +/- | 86 |
| 14-29 |  | + | 206 |
|  |  | - | 118 |
|  |  | +/- | 324 |
| 2-29 |  | + | 3663 |
|  |  | - | 835 |
|  |  | +/- | 4498 |
| 0*-2 | 11 | + | 8 |
|  |  | - | 2 |
|  |  | +/- | 10 |
| 2-14 |  | + | 4580 |
|  |  | - | 2447 |
|  |  | +/- | 7027 |
| 14-29 |  | + | 546 |
|  |  | - | 2946 |
|  |  | +/- | 3492 |
| 2-29 |  | + | 1392 |
|  |  | - | 2958 |
|  |  | +/- | 4350 |
| 0*-2 | 13 | + | 3581 |
|  |  | - | 65 |
|  |  | +/- | 3646 |
| 2-14 |  | + | 167 |
|  |  | - | 804 |
|  |  | +/- | 971 |
| 14-29 |  | + | 22 |
|  |  | - | 10 |
|  |  | +/- | 32 |
| 2-29 |  | + | 375 |
|  |  | - | 978 |
|  |  | +/- | 1353 |
| # of transcripts in analysis: | |  | 51075 |

Table S2: Numbers of enriched gene ontology terms detected through pairwise contrasts with the baseline. Numbers of enriched gene ontology (GO) terms among up- (+) and down- (-) regulated genes detected at each temperature at each time point relative to the baseline sample.

| Day | Temperature | Direction | BiNGO | ClueGO |
| --- | --- | --- | --- | --- |
| 2 | 9°C | + | 0 | 0 |
|  |  | - | 0 | 0 |
|  | 11°C | + | 10 | 0 |
|  |  | - | 20 | 0 |
|  | 13°C | + | 305 | 1545 |
|  |  | - | 10 | 0 |
| 14 | 9°C | + | 4 | 0 |
|  |  | - | 38 | 0 |
|  | 11°C | + | 334 | 1756 |
|  |  | - | 195 | 80 |
|  | 13°C | + | 16 | 0 |
|  |  | - | 40 | 1 |
| 29 | 9°C | + | 307 | 1595 |
|  |  | - | 69 | 90 |
|  | 11°C | + | 46 | 9 |
|  |  | - | 117 | 199 |
|  | 13°C | + | 32 | 4 |
|  |  | - | 47 | 28 |
| # GO terms/DE gene (+) | | | 0.20 | 0.15 |
| # GO terms/DE gene (-) | |  | 1.33 | 0.06 |

Table S3: Sample information. Sample treatment, parentage, sequencing details for RNA-seq experiment, and number and percentage of read pairs per sample retained after trimming and mapping.

| Sample ID | Day post hatch | Temperature (°C) | Tank | Father ID | Mother ID |
| --- | --- | --- | --- | --- | --- |
| D02-09-B5 | 2 | 9 | B5 | A35 | A57 |
| D02-09-B1 | 2 | 9 | B1 | A35 | A57 |
| D02-11-C2 | 2 | 11 | C2 | A35 | A57 |
| D02-09-C2 | 2 | 9 | C2 | A35 | A57 |
| D14-13-C4 | 14 | 13 | C4 | A35 | A57 |
| D14-09-B6 | 14 | 9 | B6 | A35 | A57 |
| D14-13-B2 | 14 | 13 | B2 | A35 | A57 |
| D14-09-C5 | 14 | 9 | C5 | A35 | A57 |
| D14-09-C6 | 14 | 9 | C6 | A35 | A57 |
| D14-11-B5 | 14 | 11 | B5 | A35 | A57 |
| C-5 | 0 | 9 | n/a | A35 | A57 |
| D29-09-B2 | 29 | 9 | B2 | A35 | A57 |
| D29-09-C4 | 29 | 9 | C4 | A35 | A57 |
| D29-11-C3 | 29 | 11 | C3 | A35 | A57 |
| D29-13-B3 | 29 | 13 | B3 | A35 | A57 |
| D02-13-C4 | 2 | 13 | C4 | A35 | A57 |
| D02-11-B4 | 2 | 11 | B4 | A35 | A57 |
| D02-11-C4 | 2 | 11 | C4 | A35 | A57 |
| D02-13-B3 | 2 | 13 | B3 | A35 | A57 |
| D02-13-C3 | 2 | 13 | C3 | A35 | A20 |
| C-4 | 0 | 9 | n/a | A35 | A57 |
| D14-11-B4 | 14 | 11 | B4 | A35 | A57 |
| D14-11-C3 | 14 | 11 | C3 | A35 | A57 |
| D14-13-C3 | 14 | 13 | C3 | A35 | A57 |
| D29-09-B4 | 29 | 9 | B4 | A35 | A57 |
| D29-11-B4 | 29 | 11 | B4 | A35 | A57 |
| D29-11-C4 | 29 | 11 | C4 | *1 | A05 |
| D29-13-B4 | 29 | 13 | B4 | A35 | A57 |
| D29-13-C4 | 29 | 13 | C4 | A35 | A57 |
| C-3 | 0 | 9 | n/a | *1 | A05 |

## Supplementary Figures


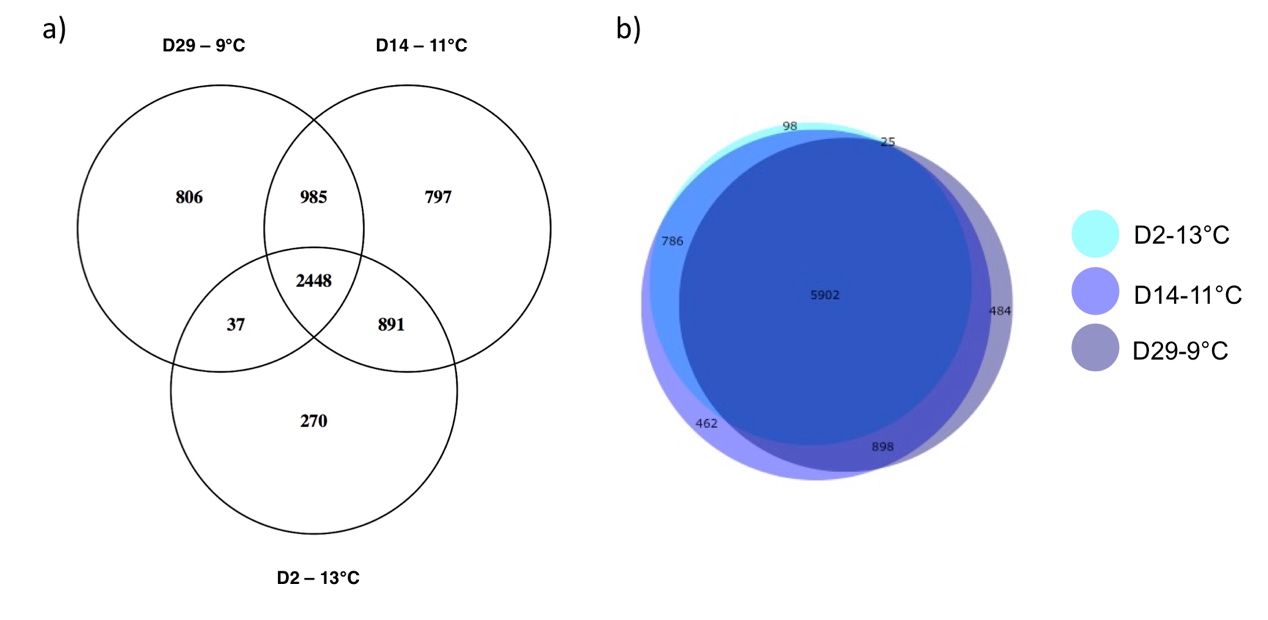
Figure S1: Overlap among peak transcriptomic responses. Numbers of differentially expressed (a) Trinity transcripts and (b) associated GO terms shared among D2-13°C, D14-11°C, and D29-9°C relative to the baseline.


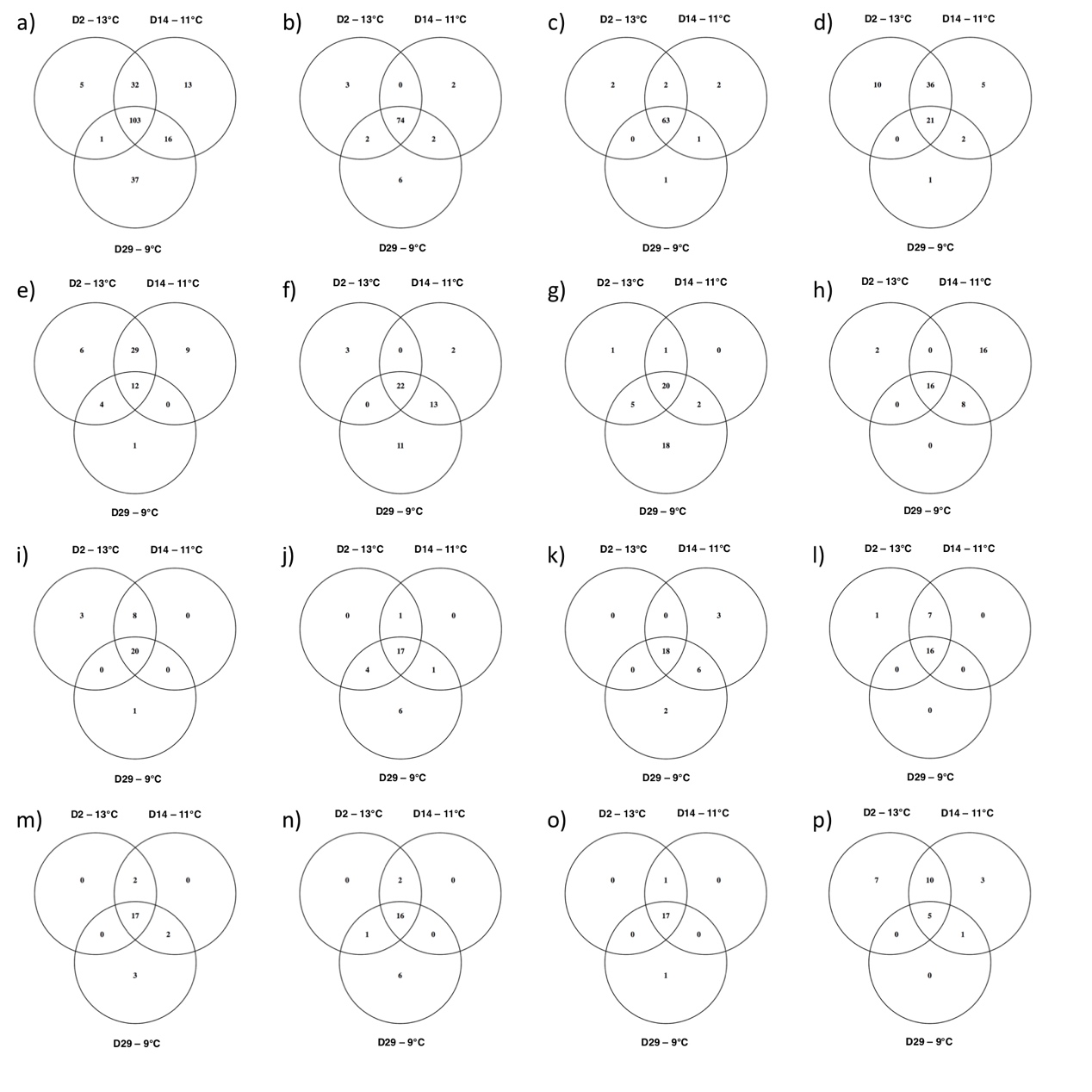
Figure S2: Overlap of gene ontology terms among peak transcriptomic responses for each gene ontology group. Venn diagrams depicting the overlap among gene ontology (GO) terms included within each manually defined GO group containing the greatest number of enriched (FDR<0.05) GO terms based on ClueGO analysis of the Trinity transcripts upregulated (FDR<0.05) at D2-13°C, D14-11°C, and D29-9°C. The GO groups are manually defined as follows, with group numbers corresponding to Supplementary data 2 given for D2-13°C, D14-11°C, and D29-9°C, respectively: (a) cell cycle and protein catabolism [210, 227+223+220, 203], (b) protein biosynthesis and metabolism [209, 226, 202], (c) protein transport and localization [208, 225, 201], (d) energy metabolism [207, 224, 193], (e) protein metabolism [206, 222, 185], (f) DNA structural modification [203, 219, 200], (g) DNA repair [204, 215, 199], (h) transcriptional regulation [192, 221, 192], (i) translational regulation [205, 218, 189], (j) RNA processing [199, 211, 196], (k) response to virus [194, 217, 195], (l) response to hormonal stimulus [202, 214, 182], (m) mitochondrial organization and mitophagy [198, 212, 190], (n) cellular organization [196, 206, 191], (o) immune response [195, 208, 187], and (p) carbohydrate metabolism [200, 209, 124].


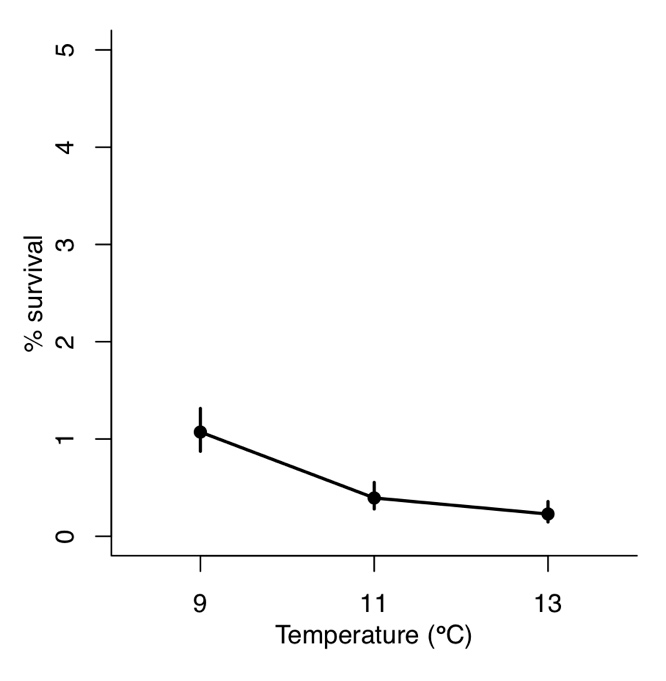


Figure S3: Thermal reaction norm for larval Skagerrak cod survival at 43 days post hatch.


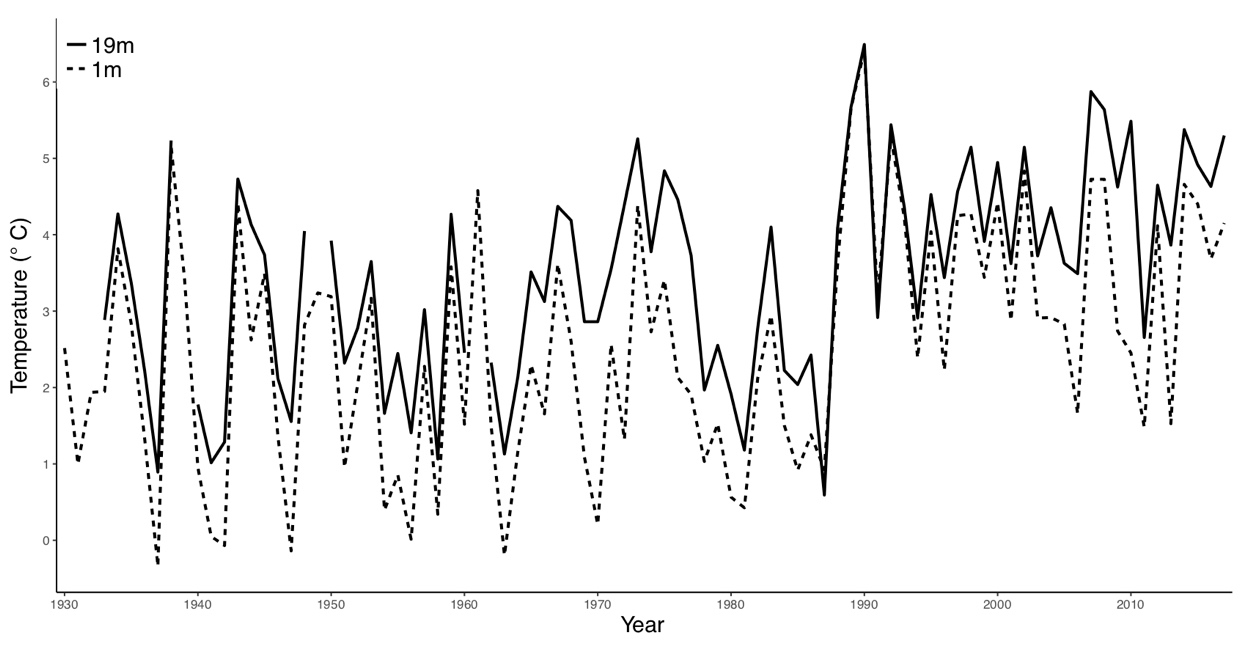
Figure S4: Sea temperature rise in study location. The average sea temperature during the peak spawning month (March) at 1m and 19m depth near the *Institute of Marine Research, Flødevigen*.

## List of Supplementary Data Files

Data S1: Enriched gene ontology terms based on genes upregulated relative to the baseline according to the Trinity pipeline and BinGO software.

<Oomen_et_al_2022_Data_S1_Trinity_BinGO_upregulated.xlsx>

Data S2: Enriched gene ontology terms based on genes upregulated relative to the baseline according to the Trinity pipeline and ClueGO software.

< Oomen_et_al_2022_Data_S2_Trinity_ClueGO_upregulated.xlsx>

Data S3: Enriched gene ontology terms based on genes downregulated relative to the baseline according to the Trinity pipeline and BinGO software.

< Oomen_et_al_2022_Data_S3_Trinity_BinGO_downregulated.xlsx>

Data S4: Enriched gene ontology terms based on genes downregulated relative to the baseline according to the Trinity pipeline and ClueGO software.

< Oomen_et_al_2022_Data_S4_Trinity_ClueGO_downregulated.xlsx>
